# Knowledge of conversion disorder by primary care physician

**DOI:** 10.1192/j.eurpsy.2022.1202

**Published:** 2022-09-01

**Authors:** I. Baati, M. Ben Abdallah, A. Arous, F. Guermazi, S. Hentati, J. Jdidi, J. Masmoudi

**Affiliations:** 1 CHU Hedi CHaker hospital Sfax Tunisia, Department Of Psychiatry (a), Sfax, Tunisia; 2 CHU Hedi CHaker hospital Sfax Tunisia, Department Of Community Health And Epidemiology, Sfax, Tunisia

**Keywords:** knowledge, Primary Care Physician, Conversion Disorder

## Abstract

**Introduction:**

Primary care physicians tend to examine patients with conversion disorder (CD) first. A good knowledge of this disorder will allow an early diagnosis and avoid unnecessary investigations for the patient.

**Objectives:**

To assess the knowledge of primary care physicians about patients with CD.

**Methods:**

We conducted a cross-sectional and descriptive study among 90 primary care physicians in Sfax (Tunisia). We used an anonymous self-questionnaire for data collection.

**Results:**

The response rate to our questionnaire was 60%. The participants’ age ranged from 25 to 70 years, with a median of 41 years. The sex ratio (M/F) was 0.92. The majority of physicians (75.9%) have practiced in the public sector. Among the respondents, 75.9% had theoretical training in CD, 14.8% had continuing medical education (CME), and 42.6% had hospital experience in a psychiatric department. The overall proportion of correct answers was 71.8%. The most recognised symptoms of CD were: dysphonia-aphonia, paresthesia or paresis. All doctors mentioned at least one criterion to distinguish CD from epileptic seizures and loss of consciousness.

**Conclusions:**

There are some gaps in primary care physicians’ knowledge of CD. Thus, we propose to reconsider the conduct of CME, to favour small group training workshops with role-playing and to improve the collaboration between the psychiatrist and the primary care physician.

**Disclosure:**

No significant relationships.

